# Central dopamine D_2_ receptors regulate plasma glucose levels in mice through autonomic nerves

**DOI:** 10.1038/s41598-020-79292-0

**Published:** 2020-12-18

**Authors:** Hiroko Ikeda, Naomi Yonemochi, Risa Mikami, Manabu Abe, Meiko Kawamura, Rie Natsume, Kenji Sakimura, John L. Waddington, Junzo Kamei

**Affiliations:** 1 Department of Pathophysiology and Therapeutics, Hoshi University School of Pharmacy and Pharmaceutical Sciences, 2-4-41 Ebara, Shinagawa-ku, Tokyo, 142-8501 Japan; 2 Department of Neurobiology, Brain Research Institute, Niigata University, Niigata, 951-8585 Japan; 3 School of Pharmacy and Biomolecular Sciences, Royal College of Surgeons in Ireland, Dublin 2, Ireland

**Keywords:** Neuroscience, Physiology, Metabolism

## Abstract

Recent evidence suggests that the central nervous system (CNS) regulates plasma glucose levels, but the underlying mechanism is unclear. The present study investigated the role of dopaminergic function in the CNS in regulation of plasma glucose levels in mice. I.c.v. injection of neither the dopamine D_1_ receptor agonist SKF 38393 nor the antagonist SCH 23390 influenced plasma glucose levels. In contrast, i.c.v. injection of both the dopamine D_2_ receptor agonist quinpirole and the antagonist l-sulpiride increased plasma glucose levels. Hyperglycemia induced by quinpirole and l-sulpiride was absent in dopamine D_2_ receptor knockout mice. I.c.v. injection of quinpirole and l-sulpiride each increased mRNA levels of hepatic glucose-6-phosphatase and phosphoenolpyruvate carboxykinase, which are the key enzymes for hepatic gluconeogenesis. Systemic injection of the β_2_ adrenoceptor antagonist ICI 118,551 inhibited hyperglycemia induced by l-sulpiride, but not by quinpirole. In contrast, hyperglycemia induced by quinpirole, but not by l-sulpiride, was inhibited by hepatic vagotomy. These results suggest that stimulation of central dopamine D_2_ receptors increases plasma glucose level by increasing hepatic glucose production through parasympathetic nerves, whereas inhibition of central dopamine D_2_ receptors increases plasma glucose level by increasing hepatic glucose production through sympathetic nerves.

## Introduction

Previous reports have indicated that plasma glucose levels are regulated by the central nervous system (CNS). For example, antipsychotic drugs such as clozapine and olanzapine are known to increase body weight and disrupt glucose metabolism^1,2^. We have previously reported that olanzapine injected intracerebroventricularly increases plasma glucose levels^3,4^ and that this hyperglycemia may involve dopamine D_2_ receptors, α_1_-adrenoceptors and histamine H_1_ receptors^5^. These results indicate that central dopamine, noradrenaline and histamine neurons play important roles in regulation of plasma glucose levels. However, the mechanisms by which central neurons regulate plasma glucose levels are unclear.

Plasma glucose levels are regulated by the balance between glucose production and glucose utilization in the whole body. It is well known that the pancreas and liver are important in regulation of plasma glucose levels. The pancreas secretes insulin and glucagon: insulin decreases plasma glucose levels by increasing glucose uptake into organs and inhibiting hepatic glucose production, whereas glucagon increases plasma glucose levels by increasing hepatic glucose production. The liver increases plasma glucose levels by glycogenolysis and gluconeogenesis and decreases plasma glucose levels by glycogenesis and glycolysis^6^. In addition, the functions of the pancreas and the liver are known to be regulated by autonomic nerves. Stimulation of sympathetic nerves increases glucagon secretion and inhibits insulin secretion from the pancreas^7,8^, and increases gluconeogenesis in the liver through β_2_ adrenoceptors^9,10^. Thus, sympathetic nerves positively regulate plasma glucose levels. In contrast, stimulation of parasympathetic nerves increases insulin secretion from the pancreas^8^ and decreases gluconeogenesis and increases glycogenesis in the liver^9,11^. Thus, parasympathetic nerves play an inhibitory role in regulation of plasma glucose levels.

The hypothalamus is known as the center of energy homeostasis. It senses peripheral metabolic signals, including hormones and nutrients, and regulates glucose metabolism^12,13^. Moreover, it is reported that the hypothalamus regulates the activity of autonomic nerves. For example, sympathetic and parasympathetic nerves projecting to the pancreas are reported to be regulated by the paraventricular nucleus of hypothalamus (PVN) and lateral hypothalamus (LH), respectively^14^. In addition, previous reports have shown that the ventromedial hypothalamus (VMH) regulates the activity of hepatic sympathetic nerves, the LH regulates the activity of hepatic vagal nerves, and the PVN regulates both sympathetic and parasympathetic nerves^9,15^. Thus, the hypothalamus is thought to regulate glucose metabolism through both sympathetic and parasympathetic nerves.

We have recently reported that dopamine D_1_ and D_2_ receptors in the hypothalamus regulate feeding behavior in mice^16^, suggesting that central dopamine D_1_ and D_2_ receptors also regulate plasma glucose levels. However, the roles of dopamine receptor subtypes in regulation of plasma glucose levels are not consistent. For instance, it has been reported that a low dose of dopamine decreases plasma glucose levels but a high dose of dopamine increases plasma glucose levels^17^. Moreover, some reports have shown that stimulation of dopamine D_2_ receptors increases plasma glucose levels^18,19^, whereas other reports have shown that stimulation of dopamine D_2_ receptors decreases plasma glucose levels^20,21^. One possible reason for these discrepancies is that dopamine receptors regulate many functions related to regulation of plasma glucose levels. In fact, it is reported that dopamine D_2_/D_3_ receptors in the pancreas regulate insulin secretion^22,23^. In addition, dopamine D_2_ receptors are known to regulate the secretion of hormones such as glucocorticoids and prolactin, which regulate plasma glucose levels^24,25^. Thus, it is likely that dopamine receptors in both the CNS and the peripheral nervous system regulate plasma glucose levels through different mechanisms.

The present study focuses on dopaminergic functions in the CNS and investigated the role of central dopamine D_1_ and D_2_ receptors in regulation of plasma glucose levels. In addition, we investigated whether autonomic nerves and hepatic glucose metabolism are involved in regulation of plasma glucose levels by central dopaminergic function.

## Results

### Effect of dopamine receptor agonists and antagonists on plasma glucose levels

**Figure 1:**
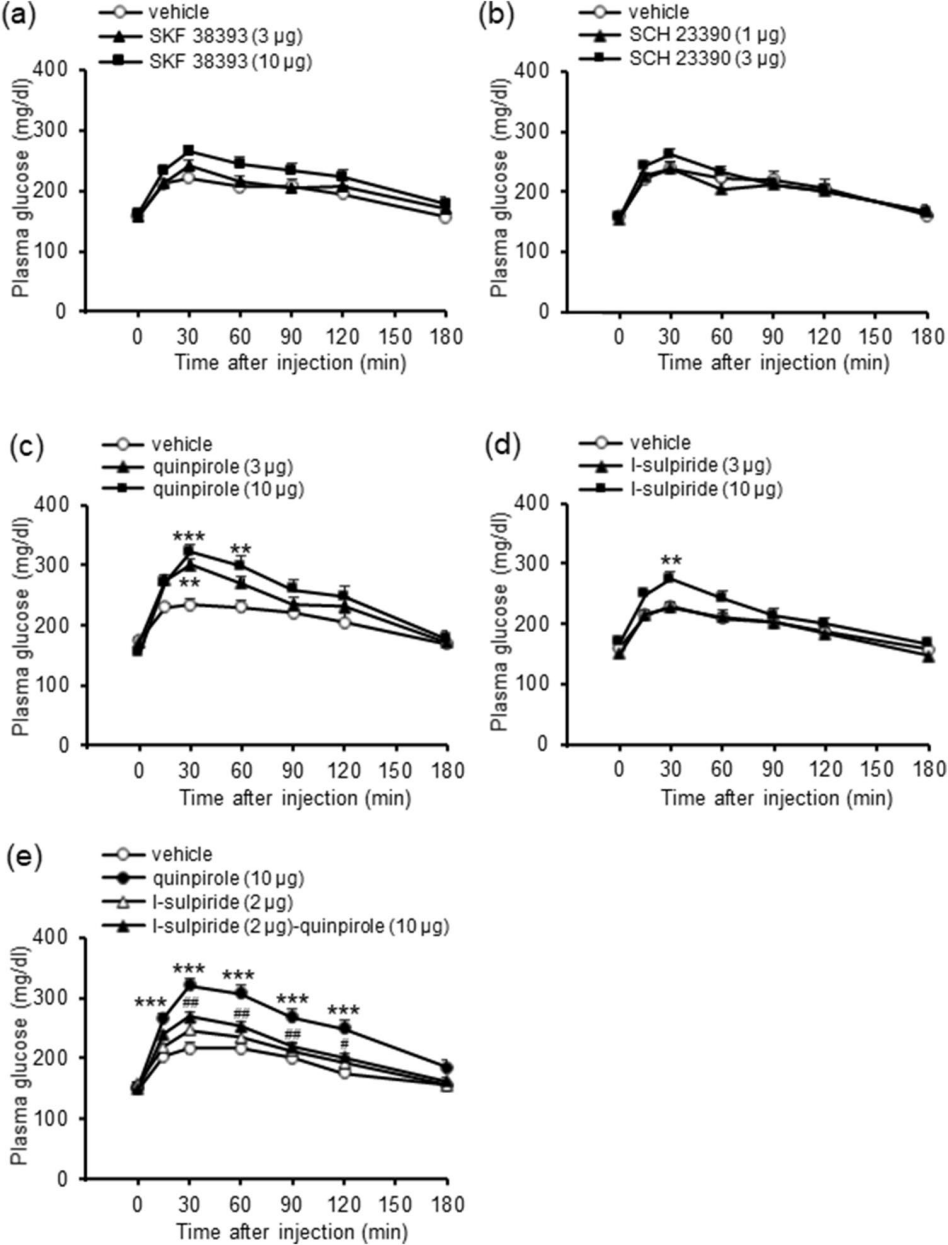
Effects of dopamine D_1_ and D_2_ receptor agonists and antagonists on plasma glucose levels in mice. (**a**) Effect of SKF 38393 (3 and 10 μg, i.c.v.) on plasma glucose levels. (**b**) Effect of SCH 23390 (1 and 3 μg, i.c.v.) on plasma glucose levels. (**c**) Effect of quinpirole (3 and 10 μg, i.c.v) on plasma glucose levels. (**d**) Effect of l-sulpiride (3 and 10 μg, i.c.v.) on plasma glucose levels. (**e**) Effect of l-sulpiride on hyperglycemia induced by quinpirole. l-Sulpiride (2 μg, i.c.v.) was co-administered with quinpirole (10 μg, i.c.v.). Each point represents the mean ± S.E.M. of 7–16 mice. **P < 0.01, ***P < 0.001 vs. vehicle group.^#^*P* < 0.05, ^##^*P* < 0.01 vs. quinpirole group.

We first examined whether central dopamine D_1_ receptors regulate plasma glucose levels in mice using i.c.v. injections of a selective dopamine D_1_ receptor agonist and a selective dopamine D_1_ receptor antagonist. Injection of neither the dopamine D_1_ receptor agonist SKF 38393 (3 and 10 μg, i.c.v.; Fig. 1a) nor antagonist SCH 23390 (1 and 3 μg, i.c.v.; Fig. 1b) influenced plasma glucose levels. These results indicate that central dopamine D_1_ receptors are not involved in regulation of plasma glucose levels.

We then examined whether central dopamine D_2_ receptors regulate plasma glucose levels using i.c.v. injections of a selective dopamine D_2_ receptor agonist and a selective dopamine D_2_ receptor antagonist. Injection of the dopamine D_2_ receptor agonist quinpirole (3 and 10 μg, i.c.v.) significantly increased plasma glucose levels (treatment: F_(2,180)_ = 4.38, *P* = 0.020; treatment × time: F_(10,180)_ = 6.61, *P* < 0.001; Fig. 1c). Injection of the dopamine D_2_ receptor antagonist l-sulpiride (3 and 10 μg, i.c.v.) also significantly increased plasma glucose levels (treatment: F_(2,220)_ = 3.27, *P* = 0.047; treatment × time: F_(10,220)_ = 3.82, *P* < 0.001; Fig. 1d). The hyperglycemia induced by quinpirole (10 μg, i.c.v.) was inhibited by a low dose of l-sulpiride (2 μg, i.c.v.) that alone did not significantly affect plasma glucose levels (treatment: F_(3,220)_ = 16.07, *P* < 0.001, treatment × time: F_(15,220)_ = 3.02, *P* < 0.001; Fig. 1e). These results indicate that both the D_2_ receptor agonist and the D_2_ receptor antagonist increase plasma glucose levels.

### Effect of dopamine D_2_ receptor agonist and antagonist on plasma glucose levels in dopamine D_2_ receptor knockout mice

**Figure 2:**
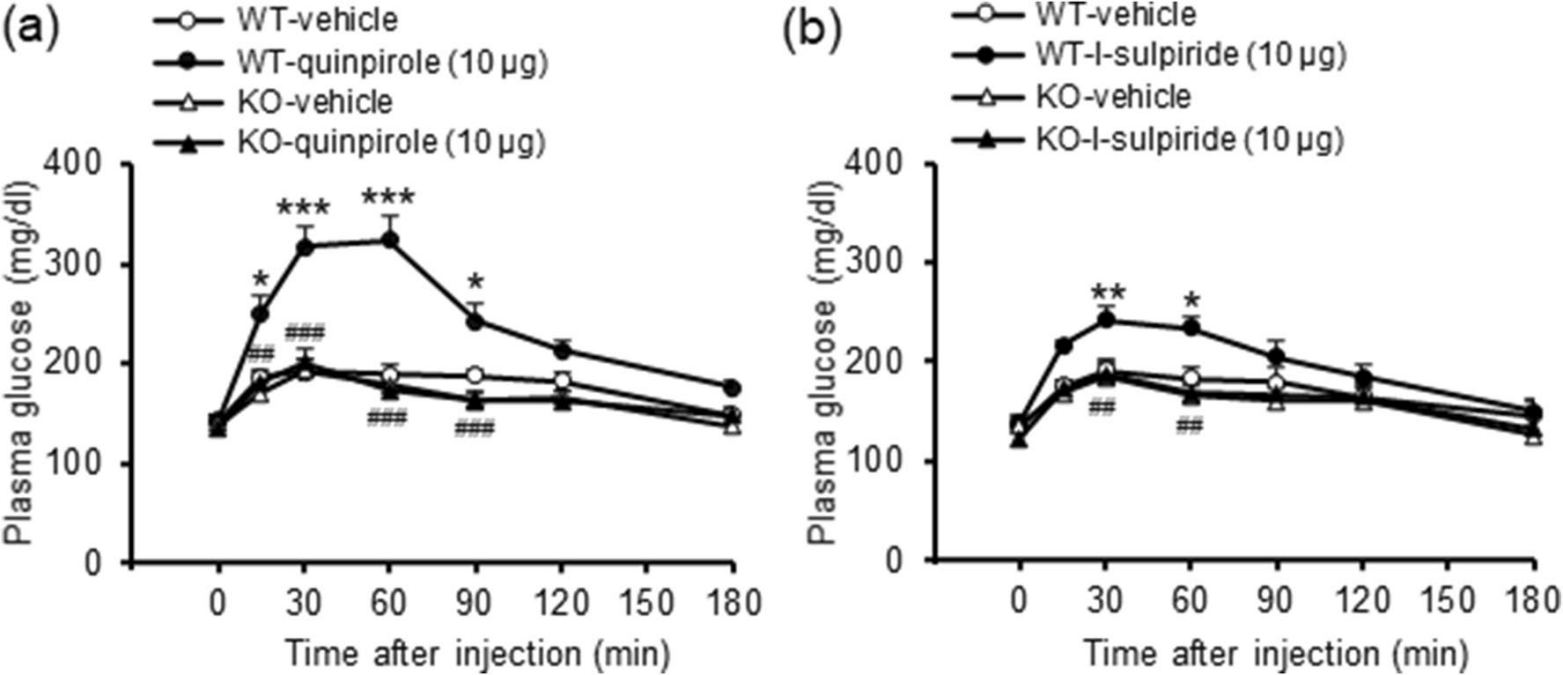
Effects of the dopamine D_2_ receptor agonist and antagonist on plasma glucose levels in dopamine D_2_ receptor knockout mice (KO). Effect of quinpirole (10 μg, i.c.v.; **a**) and l-sulpiride (10 μg, i.c.v.; **b**) on plasma glucose levels in wild-type mice (WT) and KO mice. Each point represents the mean ± S.E.M. of 6–8 mice. *P < 0.05, **P < 0.01, ***P < 0.001 vs. WT-vehicle group. ^##^*P* < 0.01, ^###^*P* < 0.001 vs. WT-quinpirole or WT-l-sulpiride group.

To confirm the involvement of dopamine D_2_ receptors in these processes, we repeated the studies using i.c.v. injections of the selective dopamine D_2_ receptor agonist and antagonist in mice with dopamine D_2_ receptor knockout. Hyperglycemia induced by quinpirole (10 μg, i.c.v.) and by l-sulpiride (10 μg, i.c.v.) was not observed in dopamine D_2_ receptor knockout mice (Fig. 2; quinpirole, treatment: F_(3,115)_ = 24.64, *P* < 0.001, treatment × time: F_(15,115)_ = 4.91, *P* < 0.001; l-sulpiride, treatment: F_(3,125)_ = 6.29, *P* = 0.003, treatment × time: F_(15,125)_ = 3.00, *P* < 0.001). These results confirm that the above actions of both the D_2_ receptor agonist and the D_2_ receptor antagonist to increase plasma glucose levels are mediated via their respective actions at dopamine D_2_ receptors.

### Effect of glucocorticoid receptor antagonist on hyperglycemia induced by dopamine D_2_ receptor agonist and antagonist

**Figure 3:**
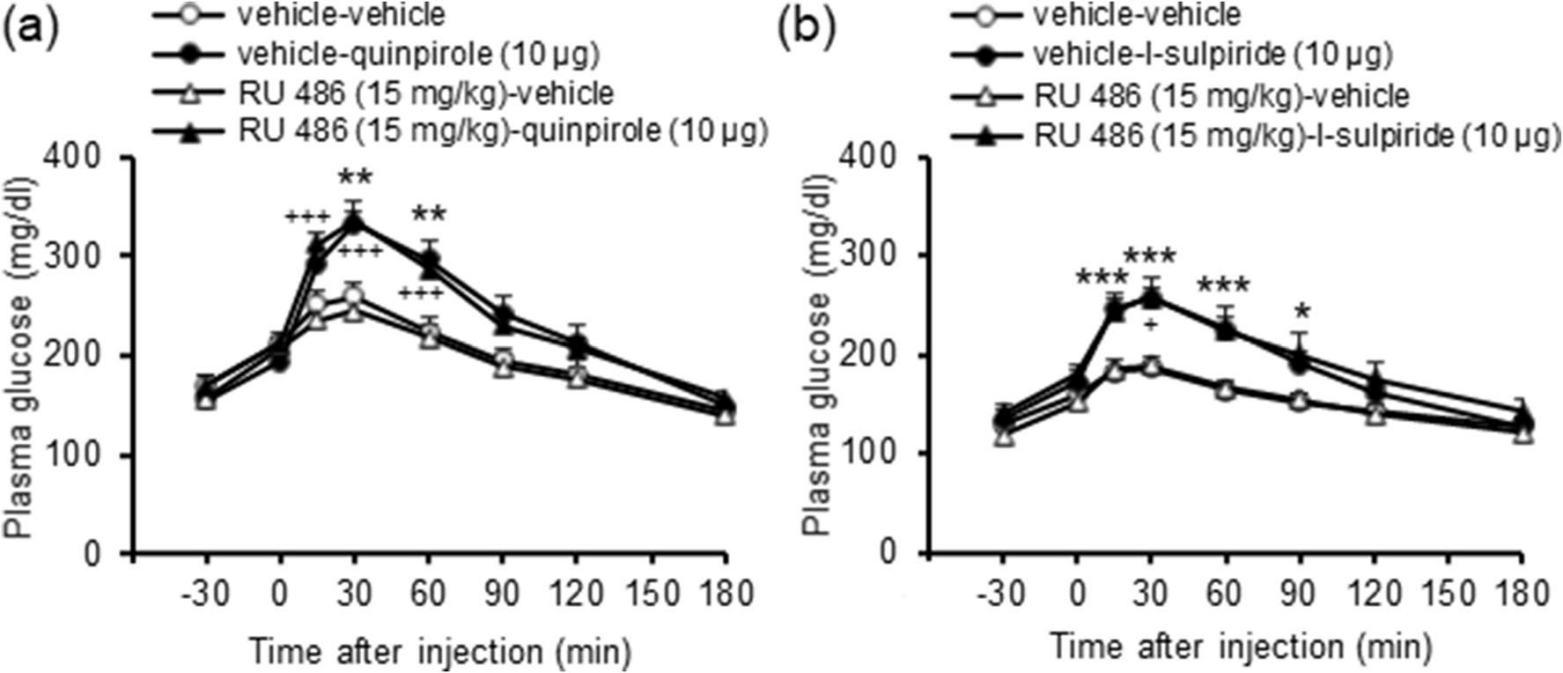
Effect of the glucocorticoid receptor antagonist on hyperglycemia induced by the dopamine D_2_ receptor agonist and antagonist. RU 486 (15 mg/kg, i.p.) was administered 30 min before injection of quinpirole (10 μg, i.c.v.; **a**) or l-sulpiride (10 μg, i.c.v.; **b**). Each point represents the mean ± S.E.M. of 7–9 mice. *P < 0.05, **P < 0.01, ***P < 0.001 vs. vehicle group. ^+^*P* < 0.05, ^+++^*P* < 0.001 vs. RU 486-vehicle group.

Since dopamine D_2_ receptors are known to regulate glucocorticoid secretion, we investigated the role of glucocorticoids in these processes by repeating the studies using i.c.v. injections of the selective dopamine D_2_ receptor agonist and antagonist following pretreatment with a glucocorticoid receptor antagonist. The glucocorticoid receptor antagonist RU 486 (15 mg/kg, i.p.) did not influence hyperglycemia induced by either quinpirole (10 μg, i.c.v.; Fig. 3a) or l-sulpiride (10 μg, i.c.v.; Fig. 3b). These results indicate that stimulation or inhibition of dopamine D_2_ receptors do not increase plasma glucose levels through regulation of glucocorticoid secretion.

### Changes in plasma glucagon and insulin levels after injection of dopamine D_2_ receptor agonist and antagonist

**Figure 4:**
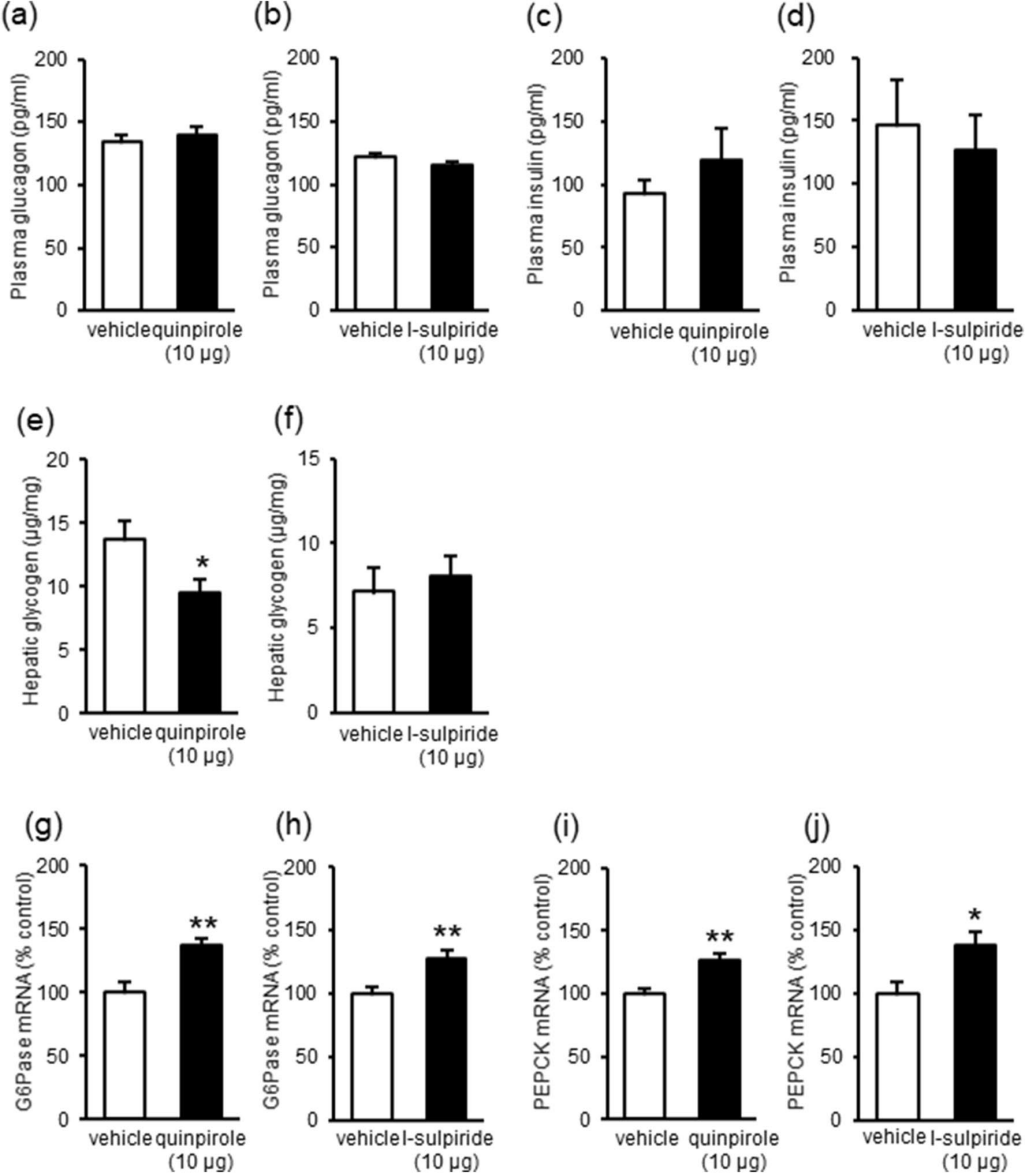
Effects of the dopamine D_2_ receptor agonist and antagonist on secretion of hormones and hepatic glucose production. (**a**–**d**) Effects of quinpirole (10 μg, i.c.v.; **a**, **c**) and l-sulpiride (10 μg, i.c.v.; **b**, **d**) on plasma glucagon (**a**, **b**) and insulin (**c**, **d**) levels. Blood samples were collected 10 min after drug injections. (**e**, **f**) Effect of quinpirole (10 μg, i.c.v.; **e**) and l-sulpiride (10 μg, i.c.v.; **f**) on hepatic glycogen levels. Liver samples were collected 30 min after drug injections. (**g**–**j**) Effect of quinpirole (10 μg, i.c.v.; **g**, **i**) and l-sulpiride (10 μg, i.c.v.; **h**, **j**) on hepatic mRNA levels of glucose-6-phosphatase (G6Pase; **g**, **h**) and phosphoenolpyruvate carboxykinase (PEPCK; **i**, **j**). Liver samples were collected 30 min after drug injections. Each point represents the mean ± S.E.M. of 6–14 mice. *P < 0.05, **P < 0.01 vs. vehicle group.

Since glucagon and insulin are known to regulate plasma glucose levels, we investigated their roles in these processes by measuring plasma glucagon and insulin levels following i.c.v. injections of the selective dopamine D_2_ receptor agonist and antagonist. Injection of neither quinpirole (10 μg, i.c.v.) nor l-sulpiride (10 μg, i.c.v.) significantly influenced plasma glucagon and insulin levels (Fig. 4a–d). These results indicate that hyperglycemia induced by stimulation or inhibition of dopamine D_2_ receptors does not involve glucagon and insulin secretion.

### Changes in hepatic glucose production after injection of dopamine D_2_ receptor agonist and antagonist

Since hepatic glucose production increases plasma glucose levels, we next investigated whether stimulation or inhibition of central dopamine D_2_ receptors increased hepatic glucose production by glycogenolysis and gluconeogenesis. Hepatic glycogen levels were decreased by quinpirole (10 μg, i.c.v.; Fig. 4e) but not by l-sulpiride (10 μg, i.c.v.; Fig. 4f). These results indicate that stimulation of central dopamine D_2_ receptors increases glycogenolysis. In addition, mRNA levels of hepatic glucose-6-phosphatase (G6Pase) and phosphoenolpyruvate carboxykinase (PEPCK), which are the key enzymes in gluconeogenesis, were increased by quinpirole (10 μg, i.c.v.; Fig. 4g,i) and l-sulpiride (10 μg, i.c.v.; Fig. 4h,j). Since G6Pase and PEPCK are rate-limiting enzymes in gluconeogenesis, these results indicate that both stimulation and inhibition of central dopamine D_2_ receptors increase gluconeogenesis. Taken together, these results indicate that stimulation or inhibition of central dopamine D_2_ receptors increases hyperglycemia through hepatic glucose production.

### Effect of adrenergic receptor antagonist on hyperglycemia induced by dopamine D_2_ receptor agonist and antagonist

**Figure 5:**
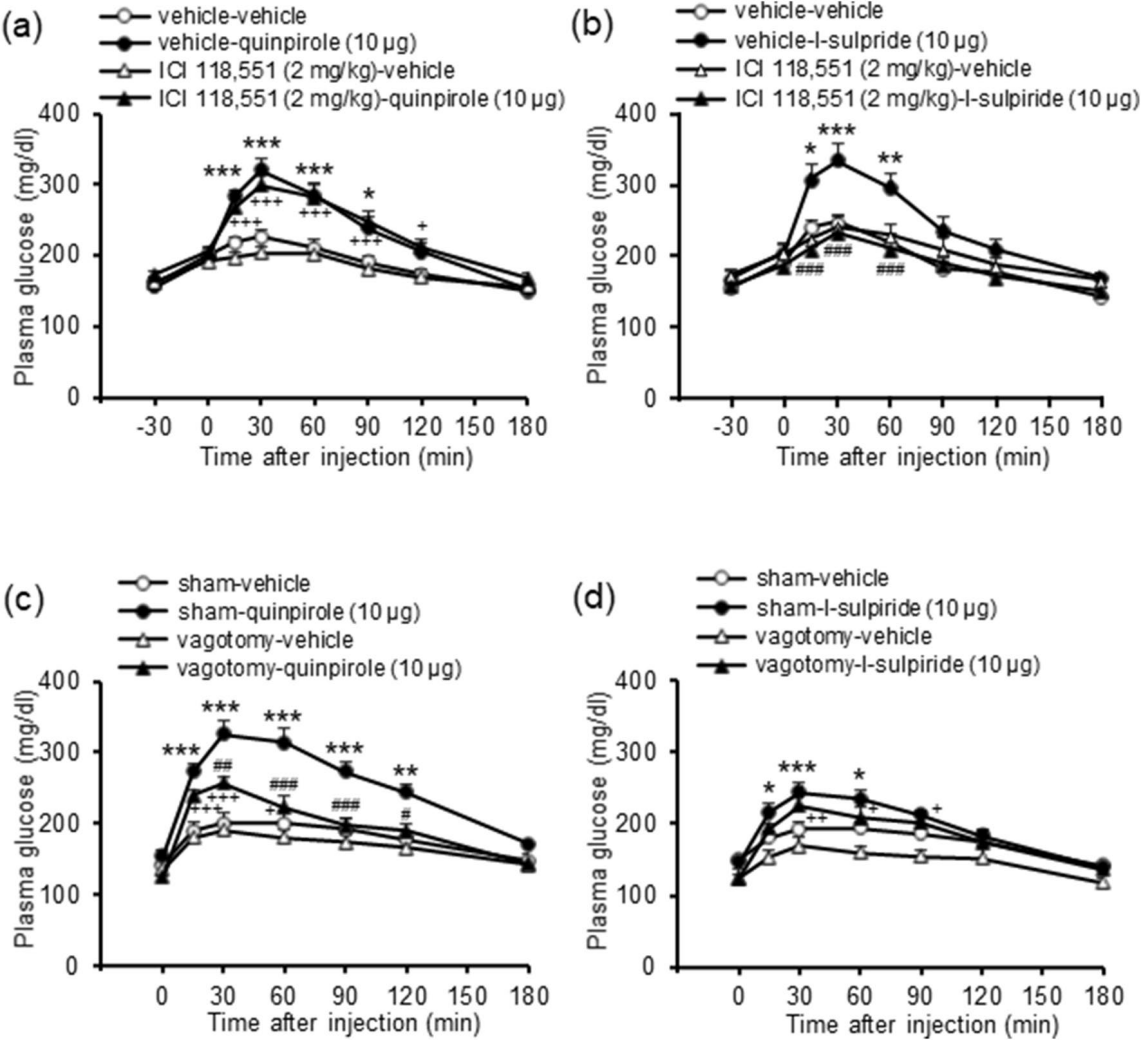
Role of sympathetic and parasympathetic nerves in hyperglycemia induced by the dopamine D_2_ receptor agonist and antagonist. (**a**, **b**) Effect of the β_2_ adrenoceptor antagonist ICI 118,551 on hyperglycemia induced by quinpirole or l-sulpiride. ICI 118,551 (2 mg/kg, i.p.) was administered 30 min before injection of quinpirole (10 μg, i.c.v.; **a**) or l-sulpiride (10 μg, i.c.v.; **b**). Each point represents the mean ± S.E.M. of 8–15 mice. *P < 0.05, **P < 0.01, ***P < 0.001 vs. vehicle-vehicle group. ^+^*P* < 0.05, ^+++^*P* < 0.001 vs. ICI 118,551-vehicle group. ^###^*P* < 0.001 vs. vehicle-l-sulpiride group. (**c**, **d**) Effect of hepatic vagotomy on hyperglycemia induced by quinpirole (10 μg, i.c.v.; **c**) and l-sulpiride (10 μg, i.c.v.; **d**). Hepatic vagotomy was conducted at least 1 week before drug injections. Each point represents the mean ± S.E.M. of 7–12 mice. *P < 0.05, **P < 0.01, ***P < 0.001 vs. sham-vehicle group. ^+^*P* < 0.05, ^++^*P* < 0.01, ^+++^*P* < 0.001 vs. vagotomy-vehicle group. ^#^*P* < 0.05, ^##^*P* < 0.01, ^###^*P* < 0.001 vs. sham-quinpirole group.

Since hepatic glucose production is regulated by sympathetic nerves through β_2_ adrenoceptors^10^, we investigated whether stimulation or inhibition of central dopamine D_2_ receptors increases plasma glucose levels through sympathetic nerves using a β_2_ adrenoceptor antagonist. The β_2_ adrenoceptor antagonist ICI 118,551 (2 mg/kg, i.p.) did not influence hyperglycemia induced by quinpirole (10 μg, i.c.v.; Fig. 5a) but significantly inhibited hyperglycemia induced by l-sulpiride (10 μg, i.c.v.; treatment: F_(3,150)_ = 6.21, *P* = 0.002, treatment × time: F_(15,150)_ = 6.80, *P* < 0.001; Fig. 5b). These results indicate that inhibition of central dopamine D_2_ receptors increases plasma glucose levels through stimulation of sympathetic nerves.

### Effects of hepatic vagotomy on hyperglycemia induced by dopamine D_2_ receptor agonist and antagonist

To investigate the involvement of parasympathetic nerves, we examined the effects of hepatic vagotomy on the ability of stimulation or inhibition of central dopamine D_2_ receptors to increase plasma glucose levels. Hepatic vagotomy significantly inhibited hyperglycemia induced by quinpirole (10 μg, i.c.v.; treatment: F_(3,135)_ = 15.53, *P* < 0.001, treatment × time: F_(15,135)_ = 8.34, *P* < 0.001; Fig. 5c). In contrast, hepatic vagotomy did not influence hyperglycemia induced by l-sulpiride (10 μg, i.c.v.; Fig. 5d). Since stimulation of parasympathetic nerves decreases plasma glucose levels, these results indicate that stimulation of central dopamine D_2_ receptors increases plasma glucose levels through inhibition of parasympathetic nerves.

## Discussion

The present study investigated the role of central dopaminergic function in regulation of plasma glucose levels.

Our results showed that i.c.v. injection of quinpirole dose-dependently increased plasma glucose levels. Surprisingly, i.c.v. injection of l-sulpiride also increased plasma glucose levels. Hyperglycemia induced by quinpirole was antagonized by a low dose of l-sulpiride. In addition, hyperglycemia induced both by quinpirole and by l-sulpiride was absent in dopamine D_2_ receptor knockout mice. Thus, these results indicate that both stimulation and inhibition of dopamine D_2_ receptors in the CNS increase plasma glucose levels. Some reports have shown that dopamine D_2_ receptors positively regulate plasma glucose levels, but other reports have shown that dopamine D_2_ receptors negatively regulate plasma glucose levels. For example, a previous report has shown that systemic injection of a low dose and a high dose of dopamine respectively increases and decreases plasma glucose levels^17^. In addition, there are reports that stimulation of dopamine D_2_ receptors increases plasma glucose levels^18,19^, whereas other reports suggest that stimulation of dopamine D_2_ receptors inhibits hyperglycemia induced by food intake or in diabetes^20,21^. Taken together, it is likely that dopamine D_2_ receptors regulate plasma glucose levels through more than one mechanism.

It is reported that systemic injection of dopamine D_2_ receptor agonists such as quinpirole and bromocriptine increase plasma glucose levels^18,19^. Moreover, hyperglycemia induced by systemic injection of quinpirole was inhibited by the centrally acting dopamine D_2_ receptor antagonist haloperidol, but not by the peripheral acting dopamine D_2_ receptor antagonist domperidone^18^. In addition, our results show that i.c.v. injection of quinpirole increases plasma glucose levels. Thus, dopamine D_2_ receptors in the CNS play an important role in regulation of plasma glucose levels, while dopamine D_2_ receptors in the pancreas are reported to regulate insulin secretion^22,23^.

Previous reports have pointed out that dopamine D_2_ receptor agonists such as bromocriptine can have a therapeutic effect in type 2 diabetes mellitus by improving glycemic control and glucose tolerance^26^. In preclinical research, there are contradictory results regarding the effect of bromocriptine. Some reports have shown that bromocriptine improves insulin-resistance and glucose intolerance^20^, whereas other reports have shown that bromocriptine induces hyperglycemia^19^. Since chronic treatment with dopamine D_2_ receptor agonists improves insulin-resistance and glucose intolerance^20,21,26^, it is likely that acute treatment with dopamine D_2_ receptor agonists increases plasma glucose levels, while chronic treatment with dopamine D_2_ receptor agonists decreases plasma glucose levels.

It is well known that dopamine D_2_ receptors inhibit hormone secretion. Among them, corticosterone is known to increase plasma glucose levels. Thus, it is possible that inhibition of dopamine D_2_ receptors increases plasma glucose levels through corticosterone secretion. However, our results showed that the glucocorticoid receptor antagonist RU 486 at an effective dose^27^ did not influence hyperglycemia induced by either quinpirole or l-sulpiride, while the β_2_ adrenoceptor antagonist ICI 118,551 completely blocked hyperglycemia induced by l-sulpiride. Thus, it appears that that corticosterone is not involved in regulation of glucose homeostasis by central dopamine D_2_ receptors.

Plasma glucose levels are regulated by hepatic glucose production^6^. Since glycogenolysis and gluconeogenesis increase plasma glucose levels, it is possible that central dopamine D_2_ receptors regulate plasma glucose levels through glycogenolysis and gluconeogenesis in the liver. The i.c.v. injection of quinpirole, but not l-sulpiride, reduced glycogen levels in the liver, suggesting that stimulation of central dopamine D_2_ receptors increases glycogenolysis. In addition, injection both of quinpirole and of l-sulpiride increased mRNA levels of G6Pase and PEPCK, which are key enzymes in gluconeogenesis. Since mRNA levels of G6Pase and PEPCK are thought to correlate with gluconeogenesis^10^, it is suggested that both stimulation and inhibition of central dopamine D_2_ receptors increase gluconeogenesis. Taken together, these results suggest that stimulation and inhibition of central dopamine D_2_ receptors increase plasma glucose levels by increasing hepatic glucose production.

It has been reported that hepatic glucose production is increased by stimulation of sympathetic nerves^9,10^. Thus, it is possible that stimulation and inhibition of dopamine D_2_ receptors increase plasma glucose levels through sympathetic nerves. Since stimulation of sympathetic nerves increases hepatic glucose production through β_2_ adrenoceptors^10^, we examined whether the β_2_ adrenoceptor antagonist ICI 118,551 influences hyperglycemia induced by quinpirole and l-sulpiride. The results showed that ICI 118,551 inhibited the hyperglycemia induced by l-sulpiride but did not influence the hyperglycemia induced by quinpirole. Since β adrenoceptors regulate glucagon and insulin secretion^28,29^, it is possible that ICI 118,551 affects glucagon and/or insulin secretion. However, the present study showed that injection of ICI 118,551 alone had no effect on plasma glucose levels, suggesting that ICI 118,551 did not affect glucagon and insulin secretion. Thus, it is unlikely that ICI 118,551 inhibits hyperglycemia induced by l-sulpiride through glucagon and insulin secretion. These findings indicate that inhibition of central dopamine D_2_ receptors increases plasma glucose levels through stimulation of sympathetic nerves.

In contrast, since stimulation of parasympathetic nerves has been reported to decrease hepatic glucose production^9,11^, it is possible that stimulation and inhibition of central dopamine D_2_ receptors increase plasma glucose level by inhibiting parasympathetic nerves. Therefore, we performed vagotomy to the liver. The results showed that hyperglycemia induced by quinpirole, but not l-sulpiride, was inhibited by vagotomy. These findings indicate that stimulation of central D_2_ receptors increases plasma glucose levels by inhibiting parasympathetic nerves.

Previous reports have suggested that activities of autonomic nerves are regulated by the hypothalamus. For instance, the VMH and the PVN regulate sympathetic nerves, whereas the LH and the PVN regulate parasympathetic nerves^14,30^. More specifically, stimulation of the LH increases activity of parasympathetic nerves to the liver, stimulation of the VMH facilitates sympathetic nerves to the liver, and the PVN integrates information from other areas of the hypothalamus and regulates both nerves^9,15^. Since dopamine D_2_ receptors are reported to exist in the hypothalamus^31^, it is possible that stimulation of dopamine D_2_ receptors in the LH and/or PVN increases plasma glucose levels through inhibition of parasympathetic nerves, whereas inhibition of dopamine D_2_ receptors in the VMH and/or PVN increases plasma glucose levels through sympathetic nerves. Future studies should further investigate both these processes and the signaling mechanisms associated with the receptor-mediated effects reported.

In conclusion, the present studies indicate that dopamine D_2_, but not D_1_, receptors in the CNS regulate plasma glucose levels. In addition, our results suggest that there are at least two mechanisms related to regulation of plasma glucose levels by central dopamine D_2_ receptors: one through sympathetic nerves and the other through parasympathetic nerves. It remains possible that dopamine D_2_ receptors in different nuclei of the hypothalamus also regulate these processes.

## Materials and methods

### Animals

Male ICR mice (age 6 weeks; body weight 27–39 g; Tokyo Animal Laboratories, Tokyo, Japan), male C57BL/6 N mice (wild type; age 11–23 weeks; body weight 23–34 g) and dopamine D_2_ receptor knockout mice (age 11–23 weeks; body weight 18–28 g) were used (see below). The mice were kept under a 12-h light/dark cycle (light on at 08:00) at a constant room temperature (24 ± 1 °C) and with free access to food and water. This study was carried out in accordance with the guidelines for the care and use of laboratory animals of Hoshi University and Niigata University, which are accredited by the Ministry of Education, Culture, Sports, Science, and Technology of Japan. The protocols were approved by the Animal Experimentation Committee of Hoshi University. All efforts were made to minimize animal suffering and to reduce the number of animals used.

### Generation of knockout mice

**Figure 6:**
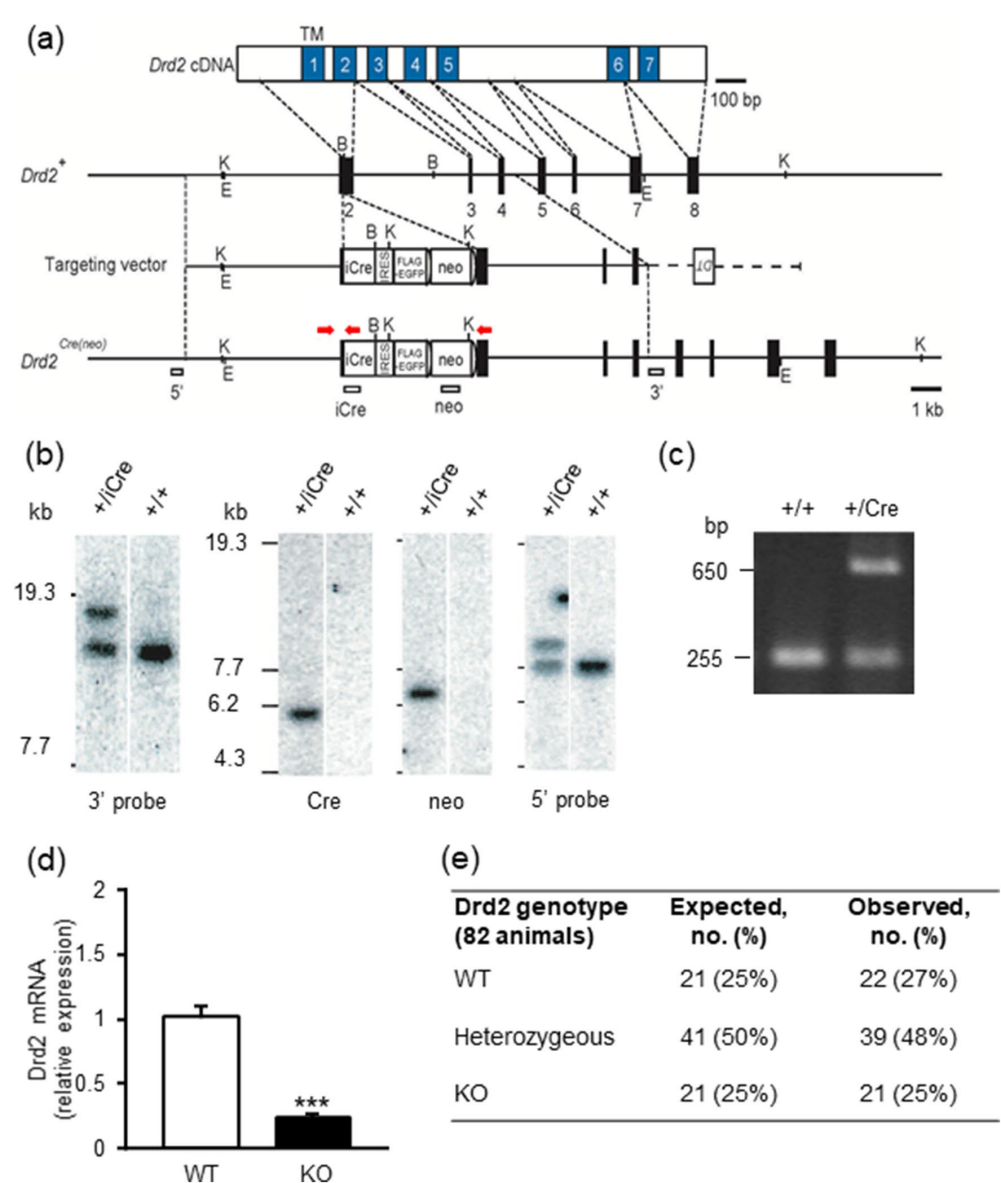
Generation of the *Drd2-Cre* mouse line. (**a**) Schema of *Drd2* cDNA, genomic DNA, targeting vector and the targeted genome. Numbered blue boxes in cDNA are transmembrane domains, and black boxes indicate coding exons. The vector was constructed to insert an improved Cre recombinase gene (*iCre*), followed by an internal ribosome entry site (*IRES*) and a *FLAG-tagged EGFP*, into the translational initiation site of the *Drd2* gene. Red arrows show primer positions for PCR genotyping. White boxes indicate probes for Southern blot analysis. Two *frt* sequences (semicircles) are attached to remove the neomycin resistance gene (Neo). B, *Bam*H1; E, *Eco*T22I; and K, *Kpn*I. (**b**) Southern blot analysis for genomic DNAs from wild-type (+ / +) and targeted (+ */iCre*) ES cells. The first panel from left shows *Eco*T22I-digested genomic DNA hybridized with 3′ probe, and the other three panels show *Kpn*I-digested DNA hybridized with Cre, *Bam*HI digested DNA, Neo and 5′ probes, respectively. Full-length images are available in Supplementary Fig. 1. (**c**) Genomic PCR genotyping of *Drd2*^+*/iCre*^ using Drd2 forward, reverse and Cre primers. Full-length images are available in Supplementary Fig. 2. (**d**) Expression of Drd2 mRNA in the hypothalamus of wild-type (WT) mice and Drd2 knockout (KO) mice. Each point represents the mean ± S.E.M. of 6–9 mice. ***P < 0.001 vs. WT group. (**e**) Observed genotypes of offspring of heterozygous intermatings, in comparison with expected genotypes calculated according to the total number of mice born (82 mice) and the expected Mendelian 1:2:1 ratio.

To produce dopamine D_2_ receptor (Drd2) protein-deficient mice, we generated knock-in mice by inserting a gene encoding the iCre protein into the initiation methionine of the gene. A total of 10.85 kbp of the gene region, containing 5.15 kbp upstream and 5.70 kbp downstream of the initiation codon in exon 2 of the *Drd2* gene, was subcloned into pDT-MC#3^32^ taken from the BAC clone RP-24-71N14. A targeting vector was constructed by inserting the iCre-IRES-Flag-EGFP-pgk-gb2neo-polyA fragment^33^ into the translational initiation site of the *Drd2* gene in frame (Fig. 6a). The vector linearized was transfected into RENKA ES cells derived from the C57BL/6 N mouse strain^34^ and recombinant clones were identified by Southern blot hybridization analysis (Fig. 6b). The recombinant ES cells were injected into eight-cell stage embryos of the CD-1 mouse strain to obtain chimeric mice. The chimeric mice were mated with C57BL/6 N mice to establish the Drd2-iCre mice (Drd2 knockout mice) on a pure C57BL/6 N genetic background. PCR-based genotyping was performed on genomic DNA extracted from mouse ear using KOD FX neo (Toyobo life science, Osaka, Japan). The following primers were used: Drd2 (forward: 5′-CTC AGC TCT GCT AGC TCT TG-3′; wild-type reverse: 5′-GCA GCA TGG CAT AGT AGT TG-3′) and Cre (5′-CAG GAA GGC CAG GTT CCT G-3′). Expected PCR products were 257 bp for the wild-type allele and 650 bp for the knockout allele (Fig. 6c). Since insertion of the gene encoding iCre into Drd2 was expected to inhibit Drd2 expression, Drd2-iCre homozygous mice can be regarded as Drd2 knockout. To confirm loss of Drd2 expression we conducted RT-PCR, which showed that Drd2 mRNA was negligible in knockout mice (Fig. 6d). Drd2 knockout mice showed the normal Mendelian ratio of offspring (1:2:1 based on 82 animals) after breeding of Drd2-iCre^+/-^ mice (Fig. 6e).

### Drugs

The drugs used were: the dopamine D_1_ receptor agonist SKF 38393 hydrochloride (Sigma-Aldrich, St. Louis, MO, USA)^16^; the dopamine D_1_ receptor antagonist R( +)-SCH 23390 hydrochloride (Sigma-Aldrich)^16^; the dopamine D_2_ receptor agonist (-)-quinpirole hydrochloride (Sigma-Aldrich)^16^; the dopamine D_2_ receptor antagonist l-sulpiride (Sigma-Aldrich)^5,16^; the glucocorticoid receptor antagonist RU 486 (Sigma-Aldrich); the β_2_ adrenoceptor antagonist ICI 118,551 hydrochloride (Sigma-Aldrich). l-Sulpiride was dissolved in a minimum volume of 0.1 N HCl, neutralized with 0.1 N NaOH to pH 6–7 and adjusted to final volume with saline^5,16^; RU 486 was dissolved in a vehicle containing 90% saline, 5% dimethylsulfoxide (Wako Pure Chemical Industries, Osaka, Japan) and 5% cremophor EL (Sigma-Aldrich); other drugs were dissolved in saline^3–5,16^.

Drugs were injected in a volume of 10 ml/kg body weight for intraperitoneal (i.p.) injection and in a volume of 4 μl for intracerebroventricular (i.c.v.) injection^3–5^. I.c.v. injection was performed as described previously^3–5,35^; the injection site was 0.0 mm anterior, 1.0 mm lateral and 3.0 mm deep from bregma and only data from mice with correctly located injections were included in the analyses^3–5^. Doses of drugs were selected according to previous reports^5,27,36,37^ and optimized in preliminary experiments.

### Measurement of plasma glucose levels

Plasma glucose levels were measured according to previous reports^3–5^. Blood samples were obtained from the tail vein. Immediately after the initial blood sample was collected, drugs were injected. RU 486 and ICI 118,551 were administered 30 min before i.c.v. injections of quinpirole or l-sulpiride. The intervals between treatments were based on preliminary experiments. Blood samples were collected 15, 30, 60, 90, 120 and 180 min after i.c.v. injections. Plasma glucose levels were determined using glucose CII-test Wako (Wako Pure Chemical Industries).

### Measurement of plasma glucagon and insulin levels

Ten min after injection of quinpirole or l-sulpiride, blood samples were collected and aprotinin (500 KIU/ml) was added. Plasma glucagon and insulin levels were determined using Glucagon EIA Kit (YK090; Yanaihara, Shizuoka, Japan; RRID: AB_2857344) and LEBIS Mouse Insulin ELISA Kit (U-type; Fujifilm Wako Shibayagi, Gunma, Japan; RRID: AB_2857341), respectively.

### Measurement of hepatic glycogen levels

The liver was collected 30 min after injection of quinpirole or l-sulpiride, then immediately frozen in liquid nitrogen and kept at -80 °C until use. Hepatic glycogen levels were determined using Glycogen Colorimetric/Fluorometric Assay Kit (K646-100; BioVision, Milpitas, CA, USA).

### Reverse transcription polymerase chain reaction (RT-PCR)

Hepatic mRNA levels were measured as described previously^3,16,38–40^: the liver was collected 30 min after drug injections, then immediately frozen in liquid nitrogen and kept at -80 °C until use; total RNA was isolated from the liver using RNAiso Plus (Takara Bio, Shiga, Japan) and reverse-transcribed to cDNA using PrimeScript RT reagent Kit (Takara Bio); the primers were mixed with buffer, dNTP mix, DNA polymerase and ultrapure water, and cDNA was added to the mixture for each reaction^3,16,38–40^. The primers were: G6Pase (forward: 5′-CGA CTC GCT ATC TCC AAG TGA-3′, reverse: 5′-GTT GAA CCA GTC TCC GAC CA-3′); PEPCK (forward: 5′-CAT ATG CTG ATC CTG GGC ATA AC-3′, reverse: 5′-CAA ACT TCA TCC AGG CAA TGT C-3′); β-actin (forward: 5′-CAT CCG TAA AGA CCT CTA TGC CAA C-3′, reverse: 5′-ATG GAG CCA CCG ATC CAC A-3′)^3^. PCR was conducted on a thermal cycler (TP650; Takara Bio) as follows: 94 °C for 4 min 30 s, followed by 33 cycles of denaturing at 94 °C for 30 s, annealing at 58 °C for 1 min, and extension at 72 °C for 1 min^3,16,38–40^. The PCR products were analyzed by electrophoresis on 1.7% agarose gels; the gel was stained with ethidium bromide, photographed with UV transillumination and intensity of the band analyzed by computer-assisted densitometry using ImageJ image-analysis software; the value for each enzyme was normalized by the respective value for β-actin, intensities of the bands obtained from drug-treated mice were compared with those of vehicle-treated mice, and the percentage of control was quantified for each sample^3,16,38–40^.

### Operation of hepatic vagotomy

Hepatic vagotomy was conducted as describe previously^41^. Mice were anesthetized with sodium pentobarbital (40 mg/kg, i.p.) and isoflurane (1 v/v% inhalation). The midline of the abdomen was incised, and the diaphragm was cut to reveal the esophageal-hepatic connections. Since this region contains a neurovascular bundle, including the hepatic branch of the vagus nerve, this branch was selectively cut. The diaphragm was sutured and then the abdomen was sutured. Mice were allowed to recover from surgery for at least 1 week.

### Statistical analysis

Results are presented as means ± S.E.M. Two-way analysis of variance (ANOVA) for repeated measures, followed by post hoc Bonferroni tests, were used to compare groups as appropriate. Student’s *t*-test was used to compare two groups as appropriate. Differences were considered statistically significant when *P* < 0.05.

## Supplementary information

Supplementary Information 1.

## Data Availability

The datasets generated and/or analysed during the current study are available from the corresponding author on reasonable request.
