# Supplementary material for: Central dopamine D2 receptors regulate plasma glucose levels in mice through autonomic nerves
Source: Sci Rep. 2020 Dec 18;10:22347. doi: 10.1038/s41598-020-79292-0 (PMC7749102; doi:10.1038/s41598-020-79292-0)

Central dopamine D_2 receptors regulate plasma glucose levels in mice through autonomic nerves

Hiroko Ikeda[1,*], Naomi Yonemochi[1], Risa Mikami[1], Manabu Abe[2], Meiko Kawamura[2], Rie Natsume[2], Kenji Sakimura[2], John L. Waddington[3], Junzo Kamei[1]

[1]Department of Pathophysiology and Therapeutics, Hoshi University School of Pharmacy and Pharmaceutical Sciences, Tokyo 142-8501, Japan
[2]Department of Neurobiology, Brain Research Institute, Niigata University, Niigata 951-8585, Japan
[3]School of Pharmacy and Biomolecular Sciences, Royal College of Surgeons in Ireland, Dublin 2, Ireland

Supplementary Figure 1

Full-length data of Figure 6b (left)

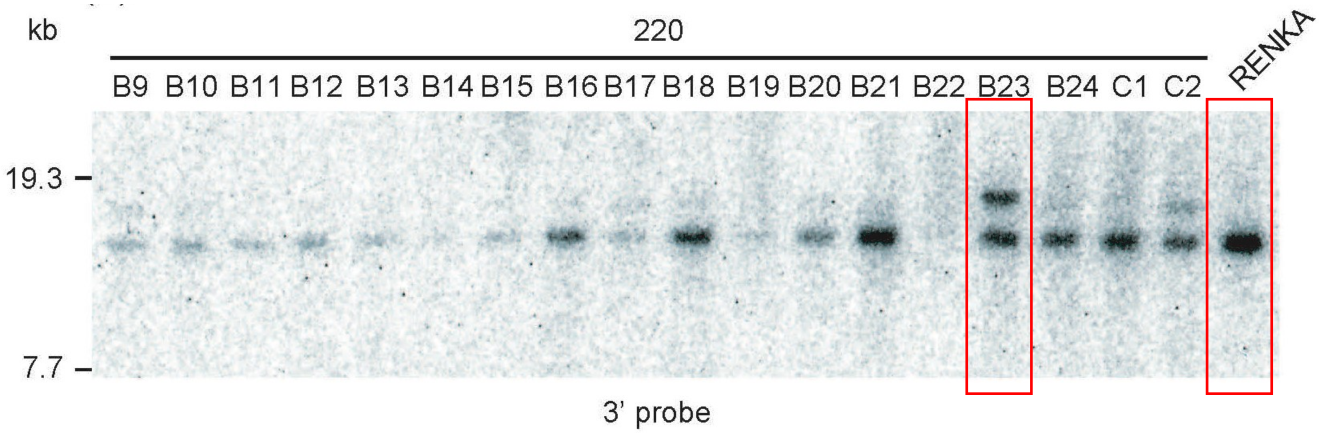

Full-length data of Figure 6b (right)

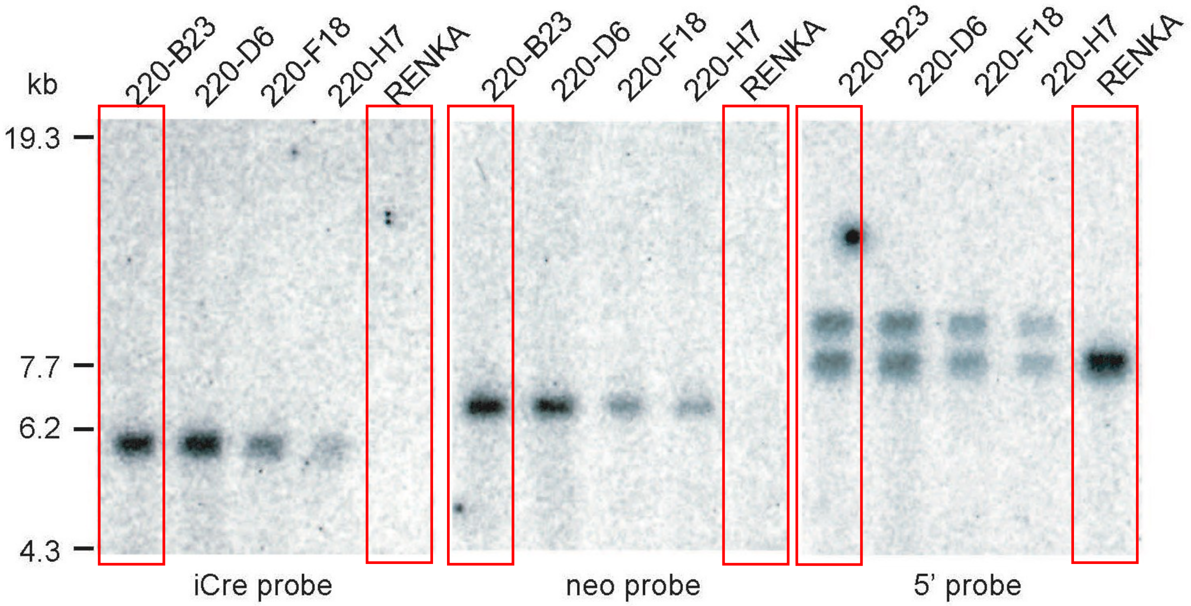

Supplementary Figure 2

Full-length data of Figure 6c

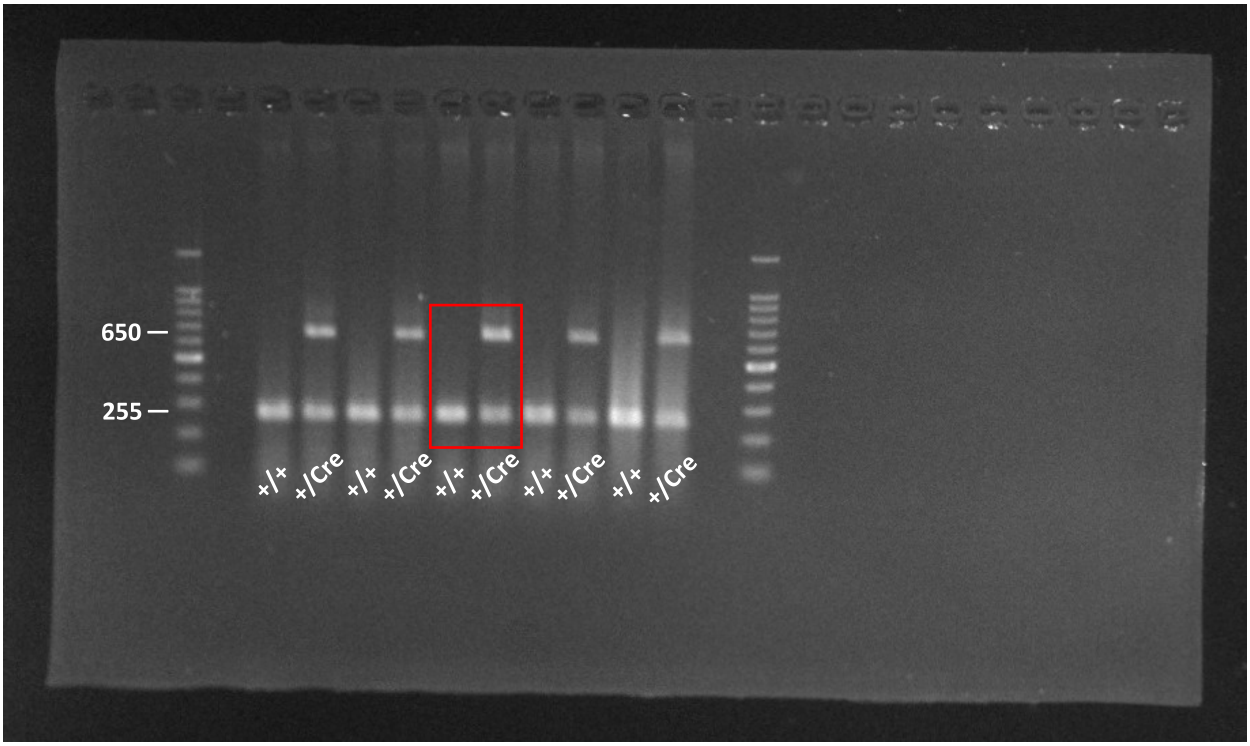

Supplement: Supplementary file 1 — Supplementary Information 1. [file 41598_2020_79292_MOESM1_ESM.pdf]
